# Transcriptome Analyses of Heart and Liver Reveal Novel Pathways for Regulating Songbird Migration

**DOI:** 10.1038/s41598-019-41252-8

**Published:** 2019-04-15

**Authors:** William J. Horton, Matthew Jensen, Aswathy Sebastian, Craig A. Praul, Istvan Albert, Paul A. Bartell

**Affiliations:** 1 Department of Animal Science, Pennsylvania State University, University Park, PA 16802 USA; 2 Bioinformatics and Genomics Program, Pennsylvania State University, University Park, PA 16802 USA; 3 The Huck Institutes of the Life Sciences, Pennsylvania State University, University Park, PA 16802 USA; 4 Department of Biochemistry and Molecular Biology, Pennsylvania State University, University Park, PA 16802 USA; 5 Center for Brain, Behavior & Cognition, Pennsylvania State University, University Park, PA 16802 USA; 6 Intercollege Graduate Degree Program in Ecology, Pennsylvania State University, University Park, PA 16802 USA

## Abstract

Many birds undertake long biannual voyages during the night. During these times of the year birds drastically reduce their amount of sleep, yet curiously perform as well on tests of physical and cognitive performance than during non-migrating times of the year. This inherent physiological protection disappears when birds are forced to stay awake at other times of the year; thus these protective changes are only associated with the nocturnal migratory state. The goal of the current study was to identify the physiological mechanisms that confer protection against the consequences of sleep loss while simultaneously allowing for the increased physical performance required for migration. We performed RNA-seq analyses of heart and liver collected from birds at different times of day under different migratory states and analyzed these data using differential expression, pathway analysis and WGCNA. We identified changes in gene expression networks implicating multiple systems and pathways. These pathways regulate many aspects of metabolism, immune function, wound repair, and protection of multiple organ systems. Consequently, the circannual program controlling the appearance of the migratory phenotype involves the complex regulation of diverse gene networks associated with the physical demands of migration.

## Introduction

Each spring and fall billions of songbirds undergo seasonal migration. Although these birds are typically diurnal, migratory flight is performed during the night. The seasonal timing of the appearance of migratory behavior is controlled by a circannual clock and shaped by daily photoperiod, while a circadian clock is responsible for the daily changes in the appearance of activity^1–3^. The circannual clock directs phenotypic changes within the body of the bird to create a so called “migratory syndrome”^4^. Although we do know that changes in some signaling molecules, such as corticosterone, adiponectin, and PACAP are part of this migratory syndrome, little is known about the molecular regulation that permit these signals to seasonally change. Other characteristics of this syndrome include, but are not limited to, fat accretion, alterations of metabolic profiles, muscle hypertrophy, increased energetic output, and the appearance of directionally oriented nocturnal wing-whirring. One consequence of the resulting nocturnal activity - commonly called Zugunruhe when displayed in captivity - is the accumulation of an extreme sleep deficit. Extreme sleep debt is typically associated with a host of health consequences, both in birds and humans (e.g. for a recent review in humans see^5^). However, birds undergoing Zugunruhe do not experience the typical maladaptive side effects associated with sleep deprivation. For example, birds exhibiting Zugunruhe perform well on cognitive tasks, although they do poorly on these tasks when forced to stay awake outside of the migratory period^6^. Birds also do not exhibit metabolic disease associated with extreme sleep deficits, such as increased incidence of diabetes and greater glucose insensitivity. Furthermore, cardiovascular disease is not only absent, but cardiovascular output is improved during migration^7^. Due to the lack of maladaptive consequences during migration, particularly in metabolic and cardiac functions, we sought to understand the mechanisms that regulate the migratory syndrome by conducting a transcriptome analysis of the heart and liver during the migratory period. To accomplish this aim, we used differential expression (DE), weighted gene co-expression network analysis (WGCNA) and pathway enrichment analyses with Ingenuity Pathway Analysis software (IPA) to analyze changes in individual genes and gene regulatory network pathways altered while maintaining the migratory state. Our results demonstrate that the phenotype associated with migratory behavior is extremely complex, involving changes in multiple genetic regulatory events and thus likely not regulated by a single switch. We present multiple regulatory pathways that may provide protective effects on the heart and liver and that can be further mined to understand mechanisms that are engaged for optimal performance during resiliency towards sleep deprivation.

## Methods

### Animals

72 White-throated sparrows (*Zonotrichia albicollis)* were captured during the fall of 2014 using mist nets in Centre County, PA (40.808749, -77.858566) and transported less than 5 km to housing at the Pennsylvania State University animal facility. All birds were housed together in a large flight cage (approximately 2.2 meters on each side) for at least six months to standardize recent life history and avoid confounds such as variations in diet and movement. Birds were given *ad-libitum* food (Mazuri small bird feed, Mazuri, Richmond, IN) and water. Initially, the light cycle was set to match the environmental photoperiod at the time of capture, but once collection was complete, the light cycle was adjusted by 30 minutes/week in a single step per week until 12.5L:11.5D was achieved. Following acclimation, animals were individually housed in small wire cages (approximately 20 × 28 × 38 cm) on racks within the larger enclosure so that migratory status could be evaluated. While flight was moderately restricted in these smaller cages, birds remained in visual and auditory contact with the remaining birds in the enclosure. In order to accelerate the transition between migratory states for tissue collection, the photoperiod was manipulated to either short (6L:18D) or long (18L:6D) days, which has previously been shown to be sufficient to shift the migratory state of birds in the laboratory. Once animals transitioned to the desired migratory state, they were returned to 12.5L:11.5D for at least one week prior to dissections, while migratory state was continually monitored (see below). This photoperiod stabilizes both the migratory and non-migratory states, and performing dissections under these conditions allowed comparisons between migratory and non-migratory conditions without confounds such as total light exposure or dissections between migratory statuses under different light conditions. All animals successfully transitioned between migratory and non-migratory states within approximately 1–2 month of altered light cycle, and were stable in the migratory condition for another 1–2 months, roughly approximating seasonal timing in the wild. There are two morphs of white-throated sparrows; to reduce variability from sex and morph differences we restricted our analyses to males of the white morph. Sex was verified by gonad inspection during dissections. All procedures followed the guidelines outlined in the NIH Care and Use of Laboratory Animals guide, and were reviewed and approved by the Pennsylvania State University IACUC Committee; Protocol #4558-1. Animals were collected under Commonwealth of Pennsylvania Wildlife Collecting Permit #32318 and USFWS Permit # MB170276-1.

### Determining migratory status

Animals were continually monitored by video and behavior was scored for a minimum of 4 nights immediately prior to tissue harvest to ascertain migratory status. Zugunruhe includes a constellation of active behaviors that occur during the night that we observed with video scoring, including increased nighttime vigilance, grooming and perch hopping. However, we have found the most robust marker that separates migratory versus non-migratory birds is a wing-whirring behavior^6,8^. Animals are unable to fly in the laboratory housing conditions; however, they are able to orient to a desired direction for migration and perform a rapid flutter of wings, which has been interpreted as a proxy for flight. This behavior is seen frequently during the night in birds in the migratory state, and never in birds that are non-migratory^6,8^. Therefore, recordings were scored in 10 minute bins for presence of or absence of wing-whirring. If the animal exhibited Zugunruhe (i.e. wing whirring) in a 10 minute bin it was given a score of one, and if it did not it was given a score of zero. In addition, fat deposit scores have previously been used to determine migratory state and were recorded during tissue collection as verification.

### Tissue collection

All tissue collection was performed under the same housing conditions (*ad libitum* food and water availability, 12.5 L:11.5D light cycle). We selected two time points across the day for tissue collection. The primary behavioral change between birds that are migrating and those that are not is seen at night when migrating birds are flying while non-migrating birds sleep. Based on preliminary studies from our lab and others^6,8^, migrating birds may sleep for a few hours at the beginning of lights out. Therefore, we chose a point in the middle of the night – Zeitgeiber time 18 (ZT18) or 18 hours after lights on - when all migratory birds were awake and displayed Zugunruhe compared to sleeping non-migratory birds. This time point captures the greatest phenotypic difference between birds in the migratory and non-migratory dispositions, but adds the potential confound of sleep status. To address this, we also collected tissue at ZT6, when both migratory and non-migratory birds are awake and feeding. Animals were decapitated and the heart and liver were quickly removed, cut into small ~5 mm diameter chunks and placed into a volume of RNAlater™ solution (ThermoFisher Scientific, Waltham, MA) 5–10 times the tissue volume. Tissue was allowed to stabilize for 1–2 days at 4 °C and then moved to storage at −20 °C until all samples were collected.

### RNA extraction, library prep and sequencing

RNA was extracted from ~50 mg of tissue using the RNeasy mini kit (Qiagen, Hilden, Germany) according to manufacturer’s instruction. DNA-digestion was performed on column, and the elution was analyzed on a Nanodrop (ThermoFisher Scientific, Waltham, MA) for concentration and Bioanalyer (Agilent Technologies, Santa Clara, CA) for RNA quality. Library preparation and sequencing was performed by the Penn State Genomics Core Facility at University Park. A barcoded library was prepared from each sample using the Illumina TruSeq Total RNA Library Prep Kit with RiboZero Gold (Illumina Inc., San Diego, CA) according to the manufacturer’s protocol. Initially, 8 samples (1 sample for each experimental group, for each tissue) were sequenced on the Illumina HiSeq2500 (Illumina Inc., San Diego, CA) in Rapid Run mode using 150 nt paired-end sequencing. These data were used for a *de novo* assembly of the transcriptome in each tissue. The remaining 5 samples per group were sequenced using the HiSeq2500 in Rapid Run mode using 100 nt single-end sequencing for transcript quantification.

### Transcriptome assembly

Raw reads were trimmed for quality and adapter content with Trimmomatic^9^ using the default settings. After trimming, the sequence quality was assessed using FastQC (Babraham Bioinformatics, Babraham, England) and compared to untrimmed data. This trimmed data was used as input for Trinity^10^ to assemble a *de novo* transcriptome for each of our tissues individually. Finally, we used TransPS^11^ to post-process and annotate the *de novo* assemblies based on the closely related, but much more complete and well annotated zebra finch genome (*Taeniopygia guttata*, release 103).

### Differential expression and weighted gene co-expression network analysis

After transcriptome assembly and annotation, we quantified expression at the gene-level by mapping the remaining 5 biological replicates to the *de novo* transcriptome using Bowtie2^12^ followed by eXpress^13^ for transcript quantification. The resulting counts were cleaned by excluding any genes with low expression (fewer than 2 counts) and then assessed for differential expression using edgeR^14^ with a 2 × 2 experimental design (time of day, migratory status, and the time x status interaction as factors) using the generalized linear model option.

As a secondary method of analysis, normalized FPKM values from eXpress were used in weighted gene co-expression network analyses^15^ (WGCNA) for each tissue. Similar to the above analysis, genes with low expression were filtered out during cleaning (any gene with FPKM < 1 in any sample). Signed networks were constructed with the following parameters: deep split of 4, minimum module size of 30, and merge cut height of 0.1. Soft thresholding power was set to 10 for the heart networks and 9 for the liver networks based on calculated scale-free topology determined for each tissue. All other parameters remained at default values.

### Pathway analysis

Output from differentially expressed genes or differentially expressed WGCNA modules were analyzed using Ingenuity Pathway Analysis software (IPA; Qiagen, Hilden, Germany) to determine over-represented canonical pathways and potential upstream regulators. The criteria for entry into analyses were FDR corrected p-value less than 0.1, and a 1.5-fold change in expression. For WGCNA modules, only hub genes were subjected to pathway analysis. Hub genes were defined as those that were highly correlated (r = 0.8 or higher) with module eigengene values.

### Statistics

All statistical tests were performed as 2-way ANOVA in R Statistics (Vienna, Austria). Factors were migratory status, zeitgeber time (ZT), and migratory × time interaction. All p-values were corrected for multiple testing using the Benjamini-Hochberg method. We considered significant effects to be those with corrected p-values of less than 0.05, whereas trends were those with p-values between 0.1 and 0.05.

## Results

### Determining migratory status

Previous studies from our lab have shown that the most robust and consistent measure of migratory status in individually housed birds is expression of wing-whirring behavior during the dark phase^6,8^. We quantified this behavior in 10 minutes bins across the entire dark phase, and found that birds we classified as migratory had significantly higher scores on this behavior compared to those who we classified as non-migratory (t = 7.93, df = 11.2, p < 0.00001; see Supplementary Fig. S1a). For additional verification, we also scored fat deposits, which have previously been used to determine migratory status^16^ (see Supplementary Fig. S1b), and while somewhat less reliable in some species, it also differentiated the migratory status confirming the behavioral classification (t = 7.2884, df = 19.996, p < 0.0000005).

### Transcriptome assembly

Each sample was sequenced to approximately 40-million reads (raw counts 40,347,295 +/− 986,413; mean +/− SE) before being trimmed for quality and adapter content, resulting in a mean count of 33,416,401 +/− 959,794 (mean +/− SE) reads that survived trimming. Trinity assembly of heart reads resulted in 921,784 transcripts, and assembly from liver reads resulted in 930,732 transcripts. These very large transcript numbers are typical for a Trinity assembly, which include all potential isoforms of each transcript. These raw transcripts were put into TransPS to condense to the gene level and annotate against the zebra finch genome, resulting in final transcriptomes of 15,129 and 14,874 annotated gene-level transcripts for heart and liver respectively. Each assembly was evaluated by BUSCO (Benchmarking Universal Single-Copy Orthologs; a measure of transcriptome completeness)^17,18^ scores. Both heart and liver achieved very high (89.7% and 90.4% respectively) completeness scores, suggesting good quality transcriptomes. Mapping reads from biological replicates was carried out with Bowtie resulting in approximately 60% mapping efficiency. Raw and processed data in the form of sequence reads and assembled transcriptomes have been deposited in GenBank and are available under the BioProject PRJNA478852 (transcriptome assembly) and GEO Series accession number GSE116989 (raw reads and quantification).

### Differential expression

**Figure 1:**
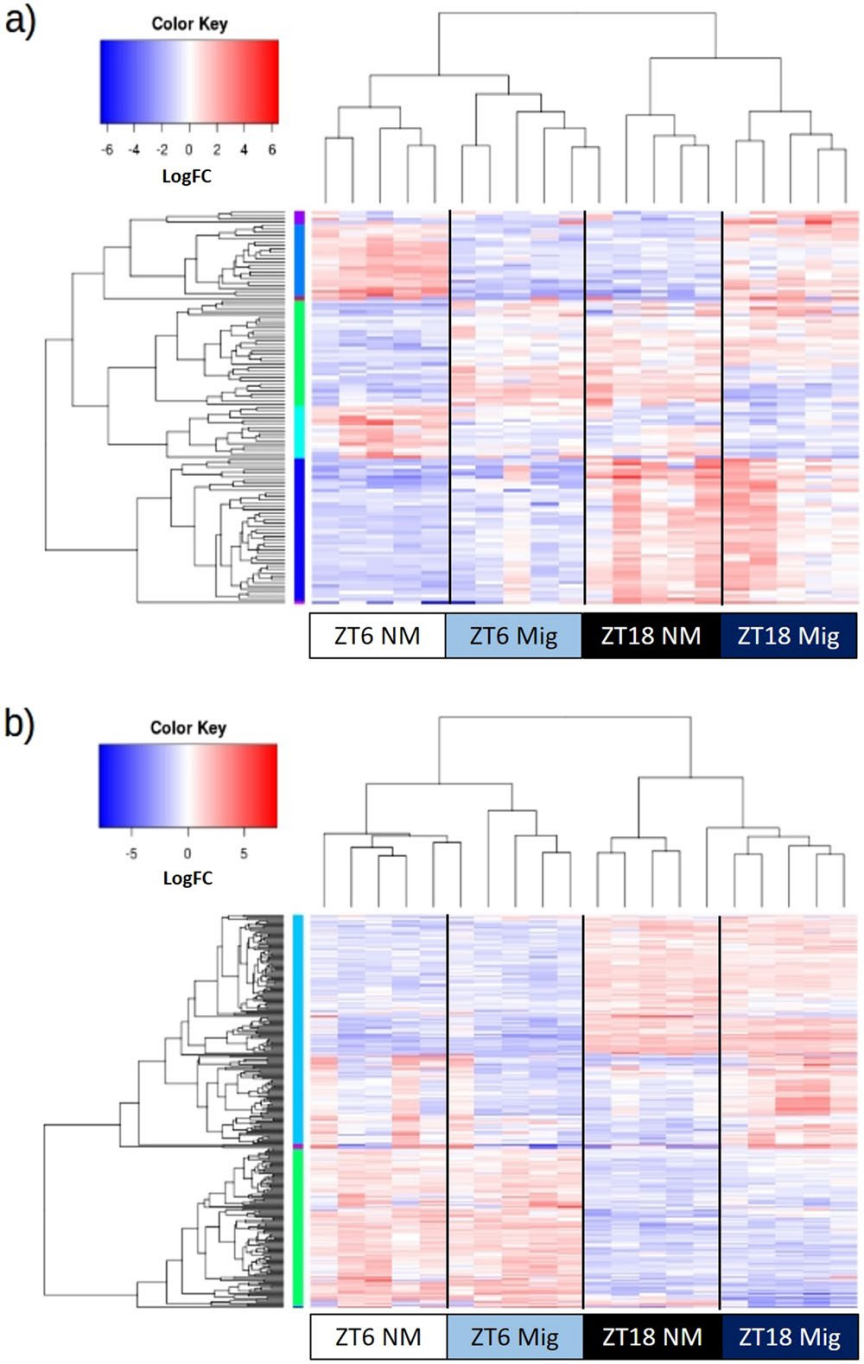
Heatmap of Differentially Expressed Genes in the Heart and Liver. Heatmaps were generated with log_2_ expression of counts normalized to transcript size and million mapped reads (FPMK values). Differentially expressed genes (DEGs) are listed along the Y-axis in the order they clustered in to as indicated by the colored line along the Y-axis. Each column contains expression values for an individual bird, with groups indicated along the X-axis, and clustering indicated by the dendogram above the figure. Deeper red colors indicate higher expression while deeper blue indicates lower expression. Panel (a) shows DEGs found in the heart, and panel (b) shows DEG observed in the liver.

After removing genes with low expression (FPKM < 1), edgeR was used to examine differential expression, defined as a 1.5-fold (or greater) difference between groups, and a p-value < 0.1 after FDR correction. These relatively lenient thresholds were chosen to allow the greatest amount of information to be included in subsequent pathway analysis steps. Heatmaps of differentially expressed genes (DEGs) across the four groups were constructed and are shown in Fig. 1a (heart) and Fig. 1b (liver). In the heart, 379 genes were differentially expressed by time of day, 684 genes by migratory status, and 69 genes showed a time × migratory status interaction. The 10 genes with the lowest corrected p-value from each factor are presented in Table 1, and the complete list is provided in Supplementary Materials S2. In the liver, 1,757 genes met criteria for differential expression in the time of day compairson, 576 genes were differentially expressed according to migratory status and six genes showed a significant interaction between the main effects. The 10 genes with the lowest corrected p-value for each factor are presented in Table 2, and the complete list is available in Supplementary Material Table S3.

**Table 1:** Top Differentially Expressed Genes in the Heart.

Gene_Symbol	logFC	logCPM	F	PValue	FDR
**Time of Day**					
AKIP1	−3.28E + 00	3.61E + 00	2.00E + 02	4.18E − 12	6.33E − 08
CPT1A	−1.83E + 00	6.73E + 00	1.67E + 02	2.33E − 11	1.76E − 07
FAM160B1	−2.21E + 00	6.08E + 00	1.40E + 02	1.17E − 10	4.66E − 07
C2H8orf22	−2.71E + 00	4.00E + 00	1.39E + 02	1.23E − 10	4.66E − 07
SLC43A2	−2.50E + 00	6.08E + 00	1.34E + 02	1.75E − 10	5.30E − 07
PER2	3.14E + 00	5.85E + 00	1.24E + 02	3.46E − 10	8.73E − 07
CTSL	−2.35E + 00	8.23E + 00	1.21E + 02	4.42E − 10	9.55E − 07
GTPBP2	−1.27E + 00	5.67E + 00	1.18E + 02	5.43E − 10	1.03E − 06
HSP90AA1	3.26E + 00	9.93E + 00	1.08E + 02	1.15E − 09	1.93E − 06
HSP90B1	1.85E + 00	8.34E + 00	9.29E + 01	4.25E − 09	6.44E − 06
**Migratory Status**					
FAM160B1	−1.89E + 00	6.08E + 00	1.04E + 02	1.56E − 09	2.37E − 05
CTSL	−2.03E + 00	8.23E + 00	9.23E + 01	4.51E − 09	2.88E − 05
LOC100229431	3.54E + 00	3.91E + 00	8.80E + 01	6.77E − 09	2.88E − 05
OXSR1	−1.13E + 00	6.79E + 00	8.68E + 01	7.61E − 09	2.88E − 05
GABARAPL1	−2.20E + 00	8.73E + 00	7.32E + 01	3.14E − 08	9.50E − 05
ARHGAP20	−1.27E + 00	5.96E + 00	6.53E + 01	7.82E − 08	1.97E − 04
BNIP3	−1.48E + 00	6.71E + 00	6.20E + 01	1.19E − 07	2.31E − 04
HMCN1	1.94E + 00	4.09E + 00	6.18E + 01	1.22E − 07	2.31E − 04
FREM2	3.02E + 00	5.74E + 00	6.07E + 01	1.39E − 07	2.34E − 04
PNPLA8	−9.50E-01	8.82E + 00	5.98E + 01	1.58E − 07	2.39E − 04
**Interaction of Time** **×** **Migratory Status**					
CTSL	2.23E + 00	8.23E + 00	5.83E + 01	1.92E − 07	1.72E − 03
GABARAPL1	2.68E + 00	8.73E + 00	5.70E + 01	2.27E − 07	1.72E − 03
FAM160B1	1.83E + 00	6.08E + 00	5.07E + 01	5.56E − 07	2.81E − 03
NMRK2	2.44E + 00	5.23E + 00	4.76E + 01	8.90E − 07	3.37E − 03
BNIP3	1.68E + 00	6.71E + 00	4.08E + 01	2.68E − 06	6.85E − 03
TOB2	1.43E + 00	8.70E + 00	4.07E + 01	2.71E − 06	6.85E − 03
SRF	2.33E + 00	7.26E + 00	3.87E + 01	3.85E − 06	8.31E − 03
FBXO32	1.93E + 00	8.07E + 00	3.77E + 01	4.60E − 06	8.71E − 03
ALPK3	1.84E + 00	8.16E + 00	3.63E + 01	6.00E − 06	1.01E − 02
EEF2K	−1.55E + 00	5.40E + 00	3.38E + 01	9.51E − 06	1.41E − 02

The top 10 differentially expressed genes for time of day, migratory status or the interaction in the heart are presented. Full lists of all differentially expressed genes can be found in Supplementary Table SI2. FC = fold change, CPM = counts per million mapped reads, FDR = corrected p-value.

**Table 2:** Top Differentially Expressed Genes in the Liver.

Gene	logFC	logCPM	F	PValue	FDR
**Time of Day**					
SCAP	3.77E + 00	5.27E + 00	4.80E + 02	7.79E − 16	1.16E − 11
PER2	5.11E + 00	5.35E + 00	4.01E + 02	4.64E − 15	3.45E − 11
CTSL	−3.92E + 00	9.94E + 00	3.12E + 02	5.48E − 14	2.72E − 10
SLC43A2	−4.05E + 00	7.55E + 00	2.57E + 02	3.53E − 13	1.31E − 09
LOC100229295	−2.74E + 00	8.30E + 00	2.40E + 02	6.84E − 13	2.00E − 09
CHD2	2.23E + 00	7.20E + 00	2.36E + 02	8.21E − 13	2.00E − 09
SLC22A5	−3.27E + 00	7.37E + 00	2.30E + 02	1.02E − 12	2.18E − 09
HAL	−3.24E + 00	9.59E + 00	2.05E + 02	3.16E − 12	5.88E − 09
CITED2	−2.82E + 00	5.16E + 00	1.95E + 02	4.93E − 12	8.00E − 09
IVNS1ABP	−4.04E + 00	1.01E + 01	1.94E + 02	5.35E − 12	8.00E − 09
**Migratory Status**					
SLC26A5	2.86E + 00	8.59E + 00	5.95E + 01	1.58E − 07	2.36E − 03
UPP2	−3.14E + 00	5.75E + 00	5.36E + 01	3.52E − 07	2.61E − 03
LOC100223441	1.26E + 00	8.55E + 00	4.78E + 01	8.31E − 07	2.67E − 03
LOC100225830	1.60E + 00	5.87E + 00	4.74E + 01	8.86E − 07	2.67E − 03
LOC100220148	3.74E + 00	7.60E + 00	4.71E + 01	9.31E − 07	2.67E − 03
UPP1	−3.23E + 00	5.13E + 00	4.62E + 01	1.08E − 06	2.67E − 03
LOC100221560	5.48E + 00	3.79E + 00	4.49E + 01	1.31E − 06	2.70E − 03
TDO2	−1.32E + 00	9.60E + 00	4.43E + 01	1.45E − 06	2.70E − 03
TTR	6.85E + 00	1.03E + 01	4.25E + 01	1.96E − 06	3.24E − 03
AK4	−1.98E + 00	4.02E + 00	3.68E + 01	5.35E − 06	7.95E − 03
**Interaction of Time** × **Migratory Status**					
UPP2	4.15E + 00	5.75E + 00	5.25E + 01	4.16E − 07	6.18E − 03
UPP1	4.25E + 00	5.13E + 00	4.59E + 01	1.13E − 06	8.39E − 03
SCAP	−1.55E + 00	5.27E + 00	4.93E + 01	2.10E − 06	1.04E − 02
PCK1	5.79E + 00	1.18E + 01	3.69E + 01	5.27E − 06	1.96E − 02
RXRG	−1.72E + 00	4.47E + 00	2.83E + 01	2.90E − 05	8.62E − 02
TAT	1.67E + 00	1.09E + 01	2.73E + 01	3.67E − 05	9.09E − 02
IRS4	1.24E + 00	6.94E + 00	2.85E + 01	5.00E − 05	1.06E-01
FABP7	−1.56E + 00	9.29E + 00	2.50E + 01	6.15E − 05	1.14E − 01
GGACT	−1.73E + 00	5.87E + 00	2.31E + 01	9.85E − 05	1.52E − 01
CDO1	1.47E + 00	1.09E + 01	2.29E + 01	1.02E − 04	1.52E − 01

The top 10 differentially expressed genes in the liver for each statistical effect are shown in this table. The complete list of all genes can be found in Supplementary Table SI3. FC = fold change, CPM = counts per million mapped reads, FDR = corrected p-value.

An abbreviated summary of the most significantly enriched pathways is shown in Table 3 (heart) and Table 4 (liver), and is fully enumerated in Supplemental Table SI4. For the main effect of time, pathways including circadian rhythm signaling and the unfolded protein response are significant. A large number of pathways are enriched for the main effect of migratory status, but broadly, there appear to be three main categories that overlap between both tissues: (1) metabolism, (2) immune function, and (3) cytoskeleton & cell growth.

**Table 3:** Top IPA Pathway Enrichment for DEGs in Heart.

Ingenuity Canonical Pathways	−log (p-value)	Ratio	z-score	Molecules
**Time of Day**				
Unfolded protein response	5.21E00	9.26E-02	NaN	HSPA8, HSP90B1, INSIG1, HSPH1, CEBPB
Mitochondrial L-carnitine Shuttle Pathway	2.34E00	9.52E-02	NaN	CPT1A, ACSBG2
eNOS Signaling	2.17E00	2.58E-02	NaN	HSPA8, AQP9, HSP90B1, HSP90AA1
Aldosterone Signaling in Epithelial Cells	2.1E00	2.47E-02	NaN	HSPA8, HSP90B1, HSPH1, HSP90AA1
Glucocorticoid Receptor Signaling	1.93E00	1.78E-02	NaN	HSPA8, HSP90B1, HSP90AA1, PCK1, CEBPB
**Migratory Status**				
T Cell Receptor Signaling	8.71E00	1.18E-01	NaN	PTPRC, CD247, CD28, PTPN7, TXK, CARD11, RASGRP1, ZAP70, PIK3CD, CD8B, LCP2, ITK
iCOS-iCOSL Signaling in T Helper Cells	8.19E00	1.06E-01	2.714	PTPRC, CD247, CD28, CD40LG, IL2RG, CD80, ZAP70, PIK3CD, INPP5D, LCP2, IL2RB, ITK
Complement System	5.38E00	1.58E-01	1.000	ITGB2, C1QC, C1QA, C1QB, CFH, C3AR1
CD28 Signaling in T Helper Cells	4.93E00	7.32E-02	2.828	PTPRC, CD247, CD28, CD80, CARD11, ZAP70, PIK3CD, LCP2, ITK
Leukocyte Extravasation Signaling	4.64E00	5.39E-02	3.317	ITGB2, SRC, NCF1, TXK, CXCR4, RASGRP1, CXCL12, PIK3CD, NCF4, ITGA4, ITK
**Interaction of Time** × **Migratory Status**				
p38 MAPK Signaling	3.24E00	2.56E-02	NaN	SRF, PLA2G4F, EEF2K
Phospholipases	2.39E00	2.94E-02	NaN	HMOX1, PLA2G4F
Phospholipase C Signaling	2.32E00	1.22E-02	NaN	HMOX1, HDAC9, PLA2G4F
PDGF Signaling	2.22E00	2.41E-02	NaN	SRF, CAV3
p53 Signaling	2.08E00	2.04E-02	NaN	HDAC9, GADD45B

Top 5 enriched pathways in the heart as determined with IPA for differentially expressed genes. Full IPA pathway enrichment lists can be found in Supplementary Information Table SI4. The −log (p-value) indicates that the molecules listed lead to pathway enrichment over expected, and ratio and z-score indicate directionality of the pathway activity.

**Table 4:** Top IPA Pathway Enrichment for DEGs in Liver.

Ingenuity Canonical Pathways	−log (p-value)	Ratio	z-score	Molecules
**Time of Day**				
TR/RXR Activation	4.48E00	9.78E-02	NaN	PIK3R3, RXRG, ADRB1, COL6A3, NXPH2, NCOR2, PCK1, ME1, RCAN2
Ketogenesis	3.64E00	2.22E-01	NaN	BDH1, HMGCLL1, HMGCL, HMGCS1
Circadian Rhythm Signaling	3.48E00	1.43E-01	NaN	PER3, GRIN2B, BHLHE41, CRY1, PER2
Amyotrophic Lateral Sclerosis Signaling	2.56E00	6.54E-02	NaN	PIK3R3, GRIN2B, CAPN6, CACNA1D, GRIA1, GRID1, GLUL
Mitochondrial L-carnitine Shuttle Pathway	2.26E00	1.43E-01	NaN	CPT1A, ACSL5, ACSBG2
**Migratory Status**				
Salvage Pathways of Pyrimidine Ribonucleotides	6.09E00	6.42E-02	NaN	UPP2, UPP1, AK4, PLK1, TTK, NEK2, CDK1
Cell Cycle: G2/M DNA Damage Checkpoint Regulation	5.48E00	1.02E-01	−2.000	CKS2, TOP2A, CCNB2, PLK1, CDK1
Mitotic Roles of Polo-Like Kinase	4.83E00	7.58E-02	2.236	KIF23, CDC20, CCNB2, PLK1, CDK1
Pyridoxal 5′-phosphate Salvage Pathway	3.39E00	5.41E-02	NaN	PLK1, TTK, NEK2, CDK1
Role of BRCA1 in DNA Damage Response	3.3E00	5.13E-02	1.000	FANCG, RBBP8, PLK1, FANCC
**Interaction of Time** × **Migratory Status**				
TR/RXR Activation	3.6E00	2.17E-02	NaN	RXRG, PCK1
Salvage Pathways of Pyrimidine Ribonucleotides	3.45E00	1.83E-02	NaN	UPP2, UPP1
4-hydroxyphenylpyruvate Biosynthesis	2.87E00	2E-01	NaN	TAT
Glucocorticoid Receptor Signaling	2.63E00	7.12E-03	NaN	TAT, PCK1
Tyrosine Degradation I	2.42E00	7.14E-02	NaN	TAT

Top 5 enriched pathways in the liver as determined with IPA performed only on differentially expressed genes from EdgeR. Full IPA pathway enrichment lists can be found in Supplementary Information Table SI4. The listed molecules lead to the significance scores for pathway enrichment, while ratio and z-scores indicate pathway activity.

### Weighted Gene Co-Expression Network Analysis (WGCNA)

**Figure 2:**
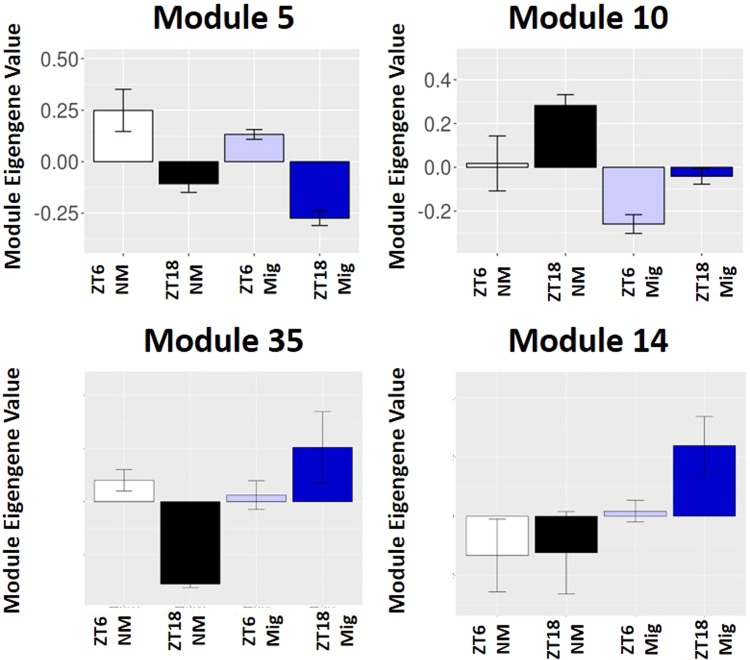
Selected WGCNA Modules. Eigengene expression for modules 5 (panel a) and 10 (panel b) from the liver, and modules 35 (**c**) and 14 (**d**) from the heart using WGCNA analysis. Non-migratory groups are indicated in monochrome and migratory groups are in blue. Lighter bars (I.E. white and light blue) indicate ZT06 while darker bars (I.E. black and dark blue) indicate ZT18. Modules 5 and 10 from the liver are both significant for main effects of time and migratory status, while modules 35 and 14 show significant main effects of migratory status. Module 35 also shows a significant time by migratory status interaction.

To reduce the dimensionality of the data and find closely related groups of genes that are differentially regulated by migration, time of day or the interaction between them, we used WGCNA^15^. Network construction in the heart resulted in genes grouped into 38 modules (See Supplemental Fig. S5(a) for dendrogram of grouping) and 28 modules in the liver (Dendrogram in Fig. S5(b)). Each of these modules were tested with a 2-way ANOVA to determine module significance. Overall, there were 18 modules with significant main effects of time of day, 14 with main effects of migratory status, and 10 modules were significant for the interaction in heart data. In the liver, 12 and 16 modules were significant for main effects of time and migratory status respectively. We did not find any modules with significant time × migratory status interactions in liver tissue. Genes with expression values that correlated highly with the composite measure of genes in the significant modules - the so-called hub genes - were analyzed with IPA to determine common pathways for each module. Complete summaries of these analyses can be found in Supplementary Tables S6 for heart and S7 for liver. However, we highlight several interesting modules here. Module 10 from the liver (Fig. 2b) showed significant main effects of both ZT time (F_(1,16)_ = 18.23, q < 0.005) and migratory status (F_(1,16)_ = 28.19, q < 0.001), but not the interaction between the two. IPA analysis revealed significant enrichment for metabolism-regulating and signaling pathways, including stearate and cholesterol biosynthesis and tryptophan degradation (Tables 5, SI7). In addition, module 35 (Fig. 2c) from the heart WGCNA analysis showed a significant main effect of migratory status (F_(1,16)_ = 15.41, q < 0.05) and the ZT time × migratory interaction (F_(1,16)_ = 23.76, q < 0.005), but no main effect of ZT time. Module 35 was enriched with immune function pathways (Tables 5, SI6). Pathways in module 35 include: IL-6-, PDGF-, and LPS-stimulated MAPK-signaling. Finally, module 14 (Fig. 2d) from the heart was significant for migratory status (F_(1,16)_ = 9.59, q < 0.05), but not ZT time or the interaction. This module was enriched for numerous pathways as seen in Table 5 (and SI6), but most prominent among them are several cytoskeletal and growth pathways, including Rho family signaling, actin cytoskeletal signaling, and reelin signaling.

**Table 5:** IPA Pathway Enrichment for Selected WGCNA Modules.

Ingenuity Canonical Pathways	−log (B-H p-value)	Ratio	z-score	Molecules
**Liver Module 5**				
Protein Ubiquitination Pathway	6.29	0.07		NEDD4, PSMA6, UBE4B, CUL1, DNAJC3, HSPA5, DNAJA1, SKP2, HSPA8, PSMD11, HSP90B1, PSMC1, PSMC6, USP47, PSMB1, HSP90AA1, PSMD1, USP37, PSMC3
Unfolded protein response	2.92	0.13		HSPA8, SCAP, HSP90B1, DNAJC3, CANX, DNAJA2, HSPA5
Aldosterone Signaling in Epithelial Cells	2.8	0.07	−1.342	HSPA8, HSP90B1, NEDD4, PDIA3, PRKCD, DNAJC3, HSP90AA1, IRS2, ITPR1, DNAJA1, HSPA5
Tryptophan Degradation III (Eukaryotic)	1.88	0.19		ACMSD, L3HYPDH, HADH, KYNU
Ketogenesis	1.75	0.3		BDH1, HMGCL, HMGCS1
**Liver Module 10**				
Stearate Biosynthesis I (Animals)	1.9	0.11		DHCR24, PPT1, ACSL5, ELOVL6
Tryptophan Degradation III (Eukaryotic)	1.55	0.14		TDO2, HAAO, ACAT1
Superpathway of Cholesterol Biosynthesis	1.35	0.11		DHCR24, ACAT1, LBR
**Heart Module 35**				
IL-6 Signaling	1.87	0.03	1	CSNK2A2, SRF, MAP3K7, PIK3R4
EGF Signaling	1.66	0.04		CSNK2A2, SRF, PIK3R4
LPS-stimulated MAPK Signaling	1.61	0.03		SRF, MAP3K7, PIK3R4
PDGF Signaling	1.61	0.03		CSNK2A2, SRF, PIK3R4
IGF-1 Signaling	1.5	0.03		CSNK2A2, SRF, PIK3R4
**Heart Module 14**				
Signaling by Rho Family GTPases	3.5	0.06	−3.051	GNB4, ACTR2, SEPT8, ACTR3, CDC42, CYFIP1, RHOA, GNA12, SEPT7, ARPC5, VIM, IRS2, ARHGEF3, ARHGEF10, SEPT2
RhoA Signaling	3.22	0.08	−2.828	SEPT8, ACTR2, ACTR3, ABL2, GNA12, RHOA, ARPC5, SEPT7, SEMA3F, SEPT2
Hepatic Fibrosis/Hepatic Stellate Cell Activation	3.22	0.07		COL5A2, COL4A1, COL6A1, MYH9, COL6A3, COL6A2, IFNGR1, MMP2, IFNAR2, PDGFB, PDGFRB, BCL2
Axonal Guidance Signaling	3.21	0.04		ACTR2, FYN, GNA12, ARPC5, MMP2, PLXND1, PDGFB, GNB4, SEMA3A, ACTR3, CDC42, RHOA, ADAM10, PLXNB2, IRS2, PRKD3, SEMA3F, PLCD4, NRP1
Rac Signaling	2.8	0.08	−2.121	ACTR2, ACTR3, CDC42, CYFIP1, RHOA, ARPC5, MAP3K1, IRS2, NCKAP1
Integrin Signaling	2.74	0.05	−2.714	FYN, ACTR2, ACTR3, CDC42, RHOA, RALB, ARPC5, ITGAV, IRS2, ITGA1, TSPAN4, PDGFB
Actin Cytoskeleton Signaling	2.64	0.05	−2.333	ACTR2, ACTR3, MYH9, CDC42, CYFIP1, GNA12, RHOA, ARPC5, IRS2, TMSB10/TMSB4X, PDGFB, NCKAP1
Actin Nucleation by ARP-WASP Complex	2.43	0.11	−2.449	ACTR2, ACTR3, CDC42, GNA12, RHOA, ARPC5
Ephrin Receptor Signaling	2.43	0.06	−2.646	FYN, ACTR2, GNB4, ACTR3, CDC42, GNA12, RHOA, ARPC5, ADAM10, PDGFB
Molecular Mechanisms of Cancer	2.37	0.04		FYN, GNA12, RALB, CDK6, BCL2, CCND2, CDC42, RHOA, IRS2, CFLAR, ARHGEF3, CTNNB1, ARHGEF10, PRKD3, BIRC2
PDGF Signaling	2.28	0.08	0.378	ABL2, INPP5J, MAP3K1, IRS2, OCRL, PDGFB, PDGFRB
Sphingosine-1-phosphate Signaling	2.28	0.07	−0.378	GNA12, CASP2, RHOA, IRS2, PLCD4, PDGFB, ASAH1, PDGFRB
Death Receptor Signaling	2.28	0.08	0.378	RIPK1, TIPARP, CASP2, CFLAR, PARP14, BCL2, BIRC2
Fcγ Receptor-mediated Phagocytosis in Macrophages and Monocytes	2.28	0.08	−2.646	FYN, ACTR2, YES1, ACTR3, CDC42, ARPC5, PRKD3
TNFR1 Signaling	2.01	0.1	−1.342	RIPK1, CDC42, CASP2, MAP3K1, BIRC2
RhoGDI Signaling	2.01	0.05	2.646	ACTR2, GNB4, ACTR3, CDC42, GNA12, RHOA, ARPC5, ARHGEF3, ARHGEF10
Epithelial Adherens Junction Signaling	1.87	0.05		ACTR2, YES1, ACTR3, MYH9, CDC42, RHOA, ARPC5, CTNNB1
NF-κB Activation by Viruses	1.76	0.07		RIPK1, MAP3K1, ITGAV, IRS2, ITGA1, PRKD3
PTEN Signaling	1.76	0.06	1.89	INPP5J, CDC42, FOXO3, PREX2, OCRL, PDGFRB, BCL2
fMLP Signaling in Neutrophils	1.76	0.06	−1.633	ACTR2, GNB4, ACTR3, CDC42, ARPC5, IRS2, PRKD3
Ephrin A Signaling	1.76	0.08		FYN, CDC42, RHOA, ADAM10, IRS2
IL-8 Signaling	1.76	0.05	−2.121	GNB4, CCND2, GNA12, RHOA, ITGAV, IRS2, MMP2, PRKD3, BCL2
Glioblastoma Multiforme Signaling	1.76	0.05	−1.134	CDC42, RHOA, CDK6, IRS2, CTNNB1, PLCD4, PDGFB, PDGFRB
PI3K/AKT Signaling	1.76	0.06	−2.449	YWHAQ, INPP5J, FOXO3, YWHAZ, CTNNB1, OCRL, BCL2
Reelin Signaling in Neurons	1.75	0.07		FYN, YES1, IRS2, ITGA1, ARHGEF3, ARHGEF10
ERK5 Signaling	1.74	0.08		YWHAQ, IL6ST, GNA12, FOXO3, YWHAZ
D-myo-inositol (1,4,5)-trisphosphate Degradation	1.7	0.17		IMPAD1, INPP5J, OCRL
CD28 Signaling in T Helper Cells	1.69	0.05	−1.89	FYN, ACTR2, ACTR3, CDC42, ARPC5, MAP3K1, IRS2
Leukocyte Extravasation Signaling	1.67	0.04	−2.121	CD99, CDC42, RHOA, PECAM1, IRS2, ITGA1, MMP2, CTNNB1, PRKD3
Ephrin B Signaling	1.52	0.07		GNB4, CDC42, GNA12, RHOA, CTNNB1
HGF Signaling	1.38	0.05	−1	ETS1, CDC42, MAP3K1, IRS2, PRKD3, ELK3
Phospholipase C Signaling	1.38	0.04		FYN, GNB4, MARCKS, HDAC4, RHOA, RALB, ARHGEF3, PRKD3, ARHGEF10
Superpathway of D-myo-inositol (1,4,5)-trisphosphate Metabolism	1.38	0.12		IMPAD1, INPP5J, OCRL
Macropinocytosis Signaling	1.38	0.06		CDC42, RHOA, IRS2, PRKD3, PDGFB
ILK Signaling	1.38	0.04	−1.633	MYH9, LIMS2, CDC42, RHOA, VIM, IRS2, TMSB10/TMSB4X, CTNNB1
Inositol Pyrophosphates Biosynthesis	1.36	0.25		IP6K1, PPIP5K2
Semaphorin Signaling in Neurons	1.35	0.08		FYN, SEMA3A, RHOA, NRP1
Thrombin Signaling	1.35	0.04	−1.134	GNB4, GNA12, RHOA, IRS2, ARHGEF3, PRKD3, ARHGEF10, PLCD4
Protein Kinase A Signaling	1.35	0.03	0	YWHAQ, GNB4, PTPRG, RHOA, MAP3K1, YWHAZ, AKAP6, PDE4D, CTNNB1, PRKD3, PLCD4, PHKG1
Role of Tissue Factor in Cancer	1.35	0.05		FYN, YES1, CDC42, GNA12, ITGAV, IRS2
phagosome formation	1.35	0.05		MARCKS, RHOA, PLA2R1, IRS2, PRKD3, PLCD4
HER-2 Signaling in Breast Cancer	1.31	0.06		CDC42, CDK6, IRS2, MMP2, PRKD3

Pathway enrichment scores and contributing genes from each of the four selected WGCNA modules (5 and 10 from the liver and 35 and 14 from the heart) are presented. Full tables for all modules can be found in Supplementary Materials Fig. S6 (heart) and S7 (liver).

## Discussion

Behavioral changes that characterize the migratory state in birds have fascinated biologists for years. However, the physiological changes that underlie this behavioral phenotype remain poorly understood. In our study, we chose to investigate the heart and the liver because these tissues are modified during migration to meet increased cardiovascular and metabolic demands, respectively, from flight and because lack of sufficient sleep can promote cardiovascular and metabolic disorders. Recent studies have used transcriptome analysis to compare gene expression within specific tissues of migrating and non-migrating or searched for polymorphisms that might be associated with migratory subpopulations^19^, however some of the findings are inconsistent across species, as described in further detail below. Because migratory birds are a non-traditional model organism, we first constructed the tools for examining wide scale gene expression. The *de novo* transcriptomes that were generated for both the liver and heart appeared to be relatively complete (as measured by BUSCO scores), and the novel TransPS tool allowed us to match our white-throated sparrow sequences to well-characterized and annotated zebra finch genes. While this step precluded detection of novel or greatly divergent genes within the sparrow, it provided a higher quality annotation than would otherwise have been possible. Because the zebra finch is a non-migratory species, it is possible that by annotating via the zebra finch genome we may have missed genes completely novel for migration. However, evolution tends to be conservative, and thus it is more likely that existing genes evolved the additional function of migration, which would still be captured with the zebra finch annotation.

Unsurprisingly, we found that pathways regulating many aspects of metabolic activity were differentially enriched between migratory states, in particular for fatty acid and carbohydrate metabolism. These findings were consistent with Singh *et al*.^20^ and Sharma *et al*.^21^ who also found wide scale changes in gene transcripts regulating metabolic pathways for lipids, carbohydrates and protein in the black headed bunting. Previous behavioral data show that prior to migration, birds become hyperphagic and increase fat stores, and that failure to do so restricts the ability to enter the migratory state^22,23^. This accreted fat is then used as an energy source during migration (Reviewed in Guglielmo, 2018)^24^, and our data are consistent with increased fatty acid utilization. However, in addition to differences in fat metabolism, we found enzymes responsible for amino acid metabolism, such as tyrosine aminotransferase, and nucleic acid salvage, such as uridine phosphorylase 1 & 2, were greatly upregulated during migration. These pathways present a mechanism by which tissues within the body can be catabolized for energy to fuel the oversized energetic demands of long-distance flight. Module 10 in the liver was heavily enriched for pathways controlling metabolism of carbohydrates, nucleic acids, lipids, and amino acids. Fudickar *et al*. similarly found differences in genes responsible for fatty acid and carbohydrate metabolism between resident and migratory dark eyed juncos^25^. These processes have implications not only for fueling flight, but also for influencing other uncovered pathways. For example, degradation of the amino acid tryptophan can induce the kyneurin signaling pathway, a process leading to immune tolerance and further changes in metabolism such as increased NAD+ synthesis^26^. NAD+ is an important co-factor for energy generation via glycolysis and lipolysis^27^. Interestingly, WGCNA module 5 in the liver shows that pathways for ketogenesis are enriched during migration, suggesting that birds may be fueling their journey, in part, by producing and burning ketones, most likely derived from fatty acid stores. Conversion of fatty acids to ketones is important as the neurons in the brain cannot metabolize fatty acids directly^28^ and the inflammasome of the immune system can be downregulated by the main ketone metabolite β-hydroxybutyrate^29^.

In the general category of cell signaling, we see several interesting pathways associated with the migratory phenotype. Although the literature on signaling changes that occur during migration is limited, our data are consistent with alterations in glucocorticoid signaling^30^ and adiponectin signaling^8^. Interestingly, we find previously unreported alterations in downstream pathways involved in mediating the effects of thyroid hormone, retinoic acid, mineralcorticoids and prolactin. Migratory behavior in Swainson’s thrushes was also strongly correlated with differential expression of gene transcripts involved in retinoic acid and thyroid hormone signaling in the brain^31^. These findings affirm our transcriptome results, in the case of glucocorticoids and adiponectin, and also provide novel hormonal pathways to explore in regards to the regulation of migratory behavior. Another system in which changes are known to occur during migration is the immune system. For example, innate immune response to a challenge with phytohemagglutinin is reduced during migration^32^. Similarly, our data showed differential gene expression and immune-related pathway enrichment during migration. In particular, module 35 in the heart demonstrates broad changes in immune signaling during migration (Fig. 2c). The immune system can also modulate the efficiency of whole body metabolism by regulating insulin sensitivity at the site of target tissues^33^. The immune system requires significant amounts of energy to be maintained in an active state, so it may be advantageous to reduce immune function during this period of intense energy demand for flight. In general, this hypothesis is borne out in our data, where we observed widespread downregulation of proinflammatory cytokine pathways. However, there was one notable except to this overall downregulation of the immune system: those portions of the proinflammatory pathways geared towards increasing levels of scavenging and remodeling of damaged tissues appear to be upregulated. These processes are similar to those found during and after successful recovery from tissue damage due to insult, such as myocardial infarct^34^. For example, some athletes involved in extreme exercise programs may experience either “athlete’s hepatitis”^35^ or cardiac myopathies as a result of either overuse or repeated transient ischemia^36^. We propose that the immune system is modified in a novel way during migration to help remove and repair the physical damage to these tissues caused by overexertion during long distance flight. Additionally, it is intriguing that many gene pathways associated with the formation of fibrosis are significantly downregulated during migration, as fibrosis formation often occurs during tissue damage as part of the wound repair process^37^. These findings further suggest that part of the migratory phenotype is the utilization of the immune system to efficiently repair and remodel tissue as it is catabolized to fuel flight. Franchini *et al*. (2017) found that the receptor for the cytokine TGF-beta is differentially expressed in the blood of migrant and resident European blackbirds, further suggesting that investigating the role of the immune system in migration may be useful in uncovering systemic regulation of these seasonal events^38^.

One unexpected finding was the general downregulation, in the migratory group, of transcripts for cellular matrix and adhesion proteins. This group of transcripts comprised roughly 6% and 6.5% of all of the differentially expressed genes in the liver and heart, respectively. When analyzed with WGCNA, we found that module 14 in the heart was particularly enriched with pathways controlling fibrosis, cytoskeleton rearrangement, and integrin regulation. These proteins are largely responsible for maintaining the extracellular spaces and providing scaffolding for the attachment of transmembrane proteins on the cell surface^39^; our findings include changes in transcripts for molecules such as cadherins, integrins, glycoproteins and multiple types of collagens. Similarly, Johnston *et al*.^31^ and Sharma *et al*.^21^ found general downregulation of genes involved in cell adhesion and cell motility, consistent with our results^31^. We hypothesize that this downregulation of extracellular matrix proteins may help modulate cell to cell communication, cell adhesion and cellular differentiation. Regulation of extracellular matrix composition may be another mechanism that birds use to facilitate repair of tissues damaged from overuse during long distance flight in a way that does not trigger the traditional wound repair mechanisms that could lead to fibrosis or scarring. This coordinated mechanism for organ rejuvenation programed within the migratory phenotype would be necessary to avoid fibrosis or scarring since birds engage in migration twice per year and have a lifespan of 10 years or more, at least in captivity. Although the life expectancy of songbirds in the wild is only a few years, due to predation and other risks, our lab currently houses several white-throated sparrows over 11 years old.

The main focus of this study was to examine changes in RNA levels that occur during migration. One interesting aspect that we attempted to capture in our data is that while both migratory and non-migratory birds share a similar phenotype during the day (i.e. awake and foraging for food), dramatic behavioral differences are seen at night (i.e. sleep when birds are non-migratory vs. awake, navigating and flying when migrating)^6^. To examine this behavioral difference, we included two time points (one during the day and one at night) for transcriptome analysis. Consistent with previous literature, transcripts and pathways known to be a part of the core molecular circadian clock or known to be directly regulated by the molecular circadian clock are significantly different between our two time points (Reviewed in Cassone *et al*.^40^). Circadian clock and clock target genes were also found to be differentially expressed with time of day in the hypothalamus of migratory Swainson’s thrushes and the liver of black headed buntings^20^. Some of our most interesting findings are in the interaction between time of day and migratory status, which implies some aspects of clock control differ with migratory phenotype, unlike what was observed in Swainson’s thrushes. Because, the number of samples required for sufficient power to detect significant gene-level interactions is high, we likely underestimated the number of genes affected by this interaction. Nevertheless, we found 10 WGCNA modules within the heart that showed significant interactive effects. These data suggest that there are suites of genes that are differentially expressed while migrating birds are awake at night, and that these are distinct from those expressed during the day in either the migratory or non-migratory states. These suites of genes, in particular, may prove the most useful in dissecting candidates for investigating clock control of migration in future work.

In summary, the work presented here provides new insights into, and key understanding of, the endogenously generated seasonal changes in the transcriptome that occur during nocturnal migration in birds. These physiological changes include increased endurance and the ability to overcome the typically deleterious effects of sleep loss. Our data are consistent with previous findings such as increased rates and efficiency of lipid metabolism. However, we were able to extend those findings to elucidate novel molecular mechanisms, such as the transcriptional control of enzymes responsible for regulation of different metabolic pathways. Also, key amongst the morphological and physiological changes during migration are those regulated by molecular pathways involved in direct cell-to-cell signaling and adhesion, such as actin and integrins. These pathways, coupled with the extensive downregulation of many immune-related pathways, are unique. They simultaneously maintain the tissue-repair and remodeling machinery at a high efficiency for both catabolism of tissue for fueling flight, fixing damage to tissue through prolonged use and ultimately providing a scaffolding network for tissue regrowth after migration, while reducing the energetic requirements of the full immune system. Our work suggests that these processes are undertaken via previously unreported mechanisms. Taken together, our data will allow for the generation of multiple novel hypotheses on the physiological regulation of migration and will lead to a more complete understanding of the unique ability of birds to dynamically reduce sleep requirements while completing ultra-endurance events without concomitant negative consequences.

## Supplementary information

Supplementary Information

Table SI2

Table SI4

Table SI7

Table SI9

Table SI10
