# Supplementary material for: Transcriptome Analyses of Heart and Liver Reveal Novel Pathways for Regulating Songbird Migration
Source: Sci Rep. 2019 Apr 15;9:6058. doi: 10.1038/s41598-019-41252-8 (PMC6465361; doi:10.1038/s41598-019-41252-8)
Supplement: Supplementary file 1 — Supplementary Information [file 41598_2019_41252_MOESM1_ESM.docx]

**Transcriptome Analyses of Heart and Liver Reveal Novel Pathways for Regulating Songbird Migration**

**William J. Horton^1^, Matthew Jensen^2^, Aswathy Sebastian^3^, Craig A. Praul^3^, Istvan Albert^3,4^, and Paul A. Bartell^1,5,6^**

1. **Department of Animal Science, Pennsylvania State University, University Park, PA. 16802, USA**
2. **Bioinformatics and Genomics Program, Pennsylvania State University, University Park, PA. 16802, USA**
3. **The Huck Institutes of the Life Sciences, Pennsylvania State University, University Park, PA 16802, USA**
4. **Department of Biochemistry and Molecular Biology, Pennsylvania State University, University Park, PA 16802, U.S.A.**
5. **Center for Brain, Behavior & Cognition, Pennsylvania State University, University Park, PA 16802, U.S.A.**
6. **Intercollege Graduate Degree Program in Ecology, Pennsylvania State University, University Park, PA. 16802, USA**

***** Correspondence to should be addressed to Paul Bartell, [pab43@psu.edu](mailto:pab43@psu.edu)

**Figure SI1: Determining Migratory Status by Expression of Zugunruhe.**

Birds in migratory state showed significantly more episodes of Zugunruhe (A; t = 7.93, df = 11.21, p < 0.00001) and significantly greater fat deposits (B; t = 7.2884, df = 19.996, p < 0.0000005).

**Table SI2: Differentially Expressed Genes in the Heart**

Full listing of differentially expressed genes for time of day, migratory status or the interaction in heart are presented.

**Figure SI3: Volcano Plots of Differentially Expressed Genes in the Heart.**

Volcano plots of differentially expressed genes in the heart as a factor of the main effect of time of day (A), migratory status (B) or the interaction of time of day x migratory status (C). Black colored points indicate non-significant findings, while red-colored dots are significant at p<0.05.

**Table SI4: Differentially Expressed Genes in the Liver**

Full listing of differentially expressed genes for time of day, migratory status or the interaction in liver are presented.

**Figure SI5: Volcano Plots of Differentially Expressed Genes in the Liver.**

Volcano plots of differentially expressed genes in the liver as a factor of the main effect of time of day (A), migratory status (B) or the interaction of time of day x migratory status (C). Black colored points indicate non-significant findings, while red-colored dots are significant at p<0.05.

**Figure SI6: Overlap of Differentially Expressed Genes by Tissue and Effect.**

Venn diagrams showing the number of unique and common genes with effects of time of day, migratory status and the time of day x migratory status interaction. Panel A diagrams the data from the heart, and panel B shows the genes from the liver.

**Table SI7: IPA Pathway Enrichment for DEGs in Heart and Liver**

Full list of IPA enriched pathways from DEGs.

**Figure SI8: WGCNA Network Construction in Heart and Liver**

This dendrogram shows weighted gene co-expression network construction in the heart. Genes are grouped into modules, as denoted by the colored bar along the bottom, based on hierarchical clustering. The y-axis is a measure of dissimilarity based on topological overlap, while each line represents a single gene.

**Table SI9: WGCNA Summary Statistics and IPA Enrichments in Heart**

This table contains results of 2-way ANOVA (Time, Migratory Status, and Time x Migratory) statistics, as well as IPA enrichment results from the WGCNA analysis in the heart.

**Table SI10: WGCNA Summary Statistics and IPA Enrichments in Liver**

This table contains results of 2-way ANOVA (Time, Migratory Status, and Time x Migratory) statistics, as well as IPA enrichment results from the WGCNA analysis in the liver.


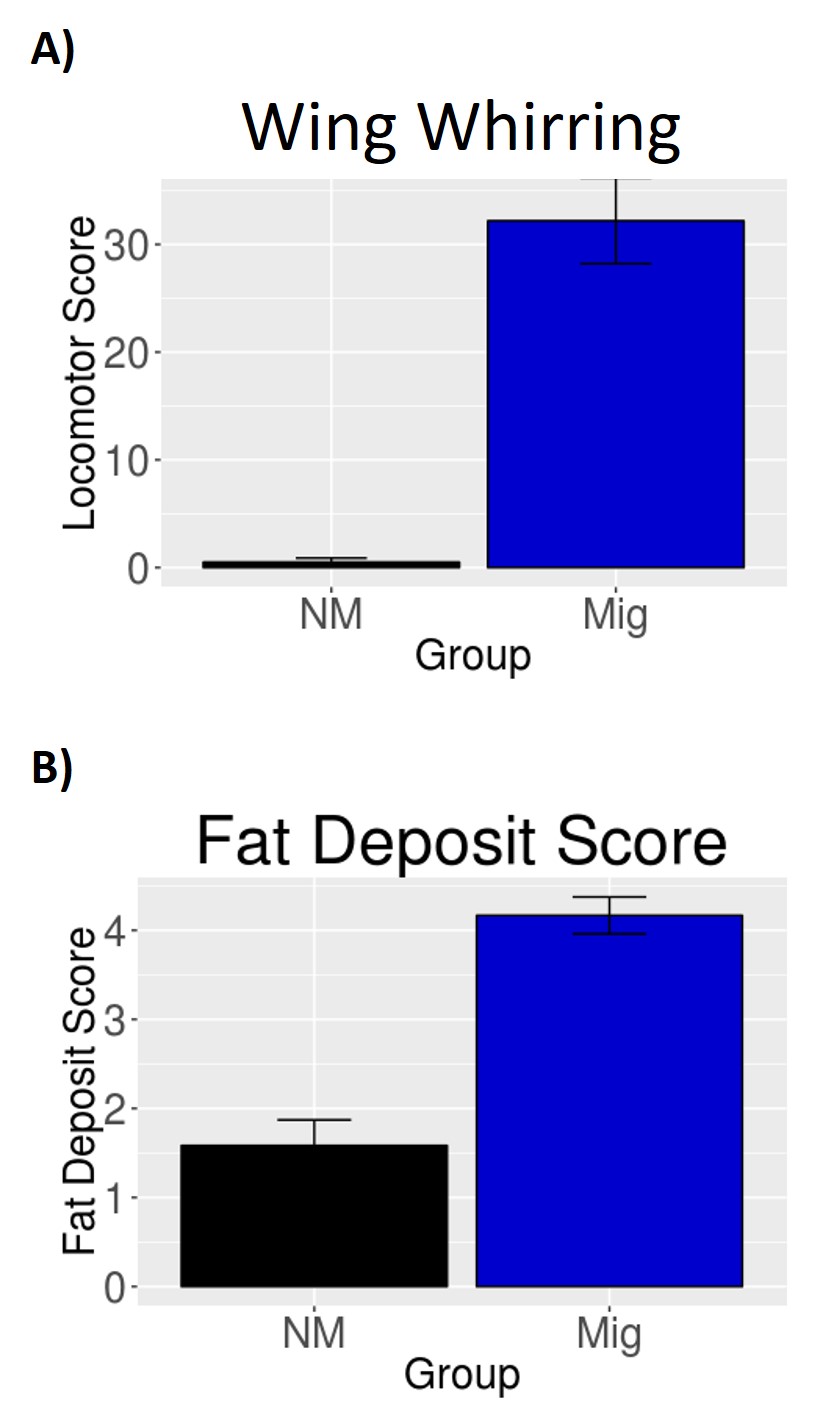


**Figure SI1: Determining Migratory Status by Expression of Zugunruhe.**

Birds in migratory state showed significantly more episodes of Zugunruhe (A; t = 7.93, df = 11.21, p < 0.00001) and significantly greater fat deposits (B; t = 7.2884, df = 19.996, p < 0.0000005).


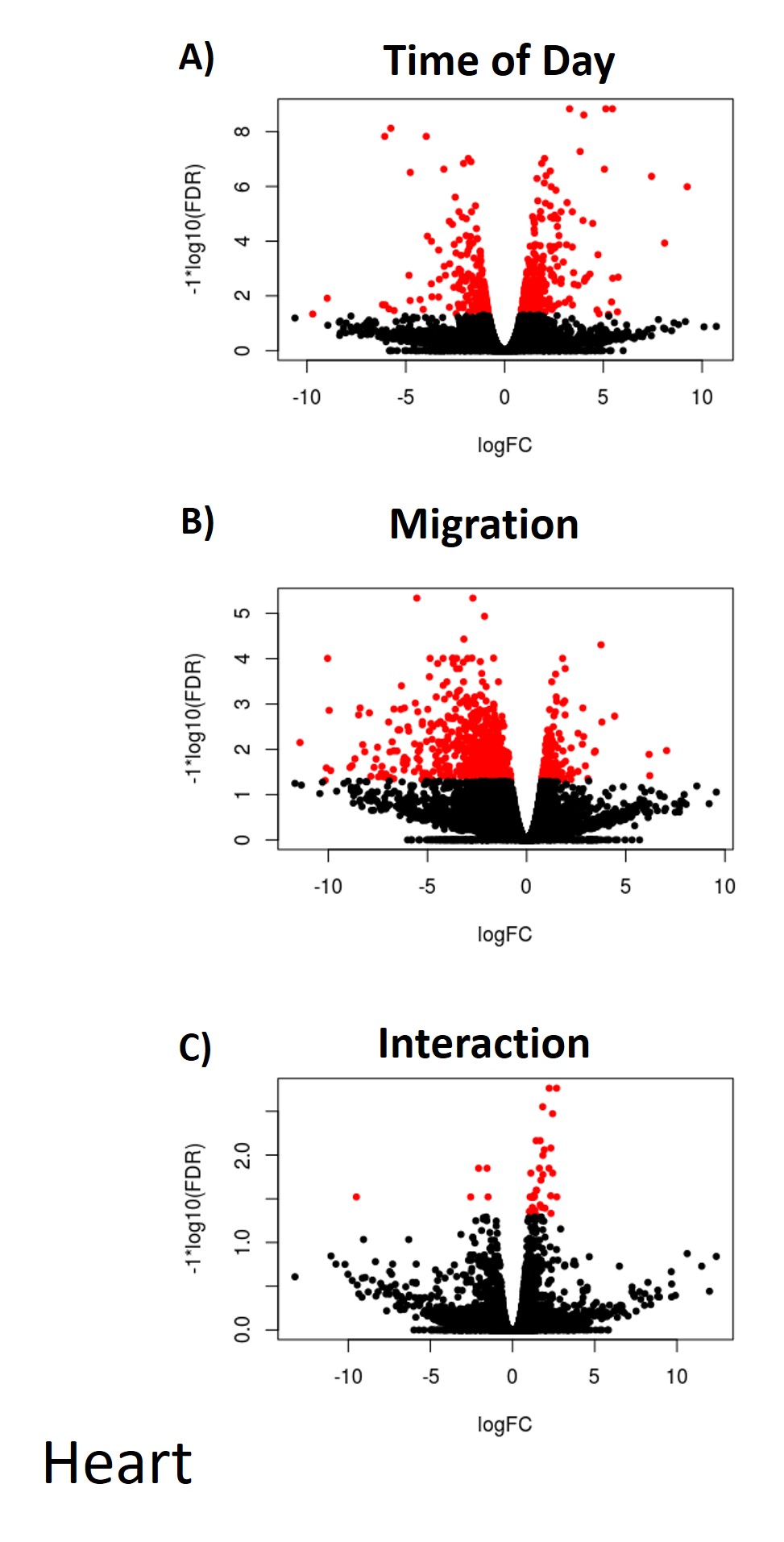


**Figure SI3: Volcano Plots of Differentially Expressed Genes in the Heart.**

Volcano plots of differentially expressed genes in the heart as a factor of the main effect of time of day (A), migratory status (B) or the interaction of time of day x migratory status (C). Black colored points indicate non-significant findings, while red-colored dots are significant at p<0.05.


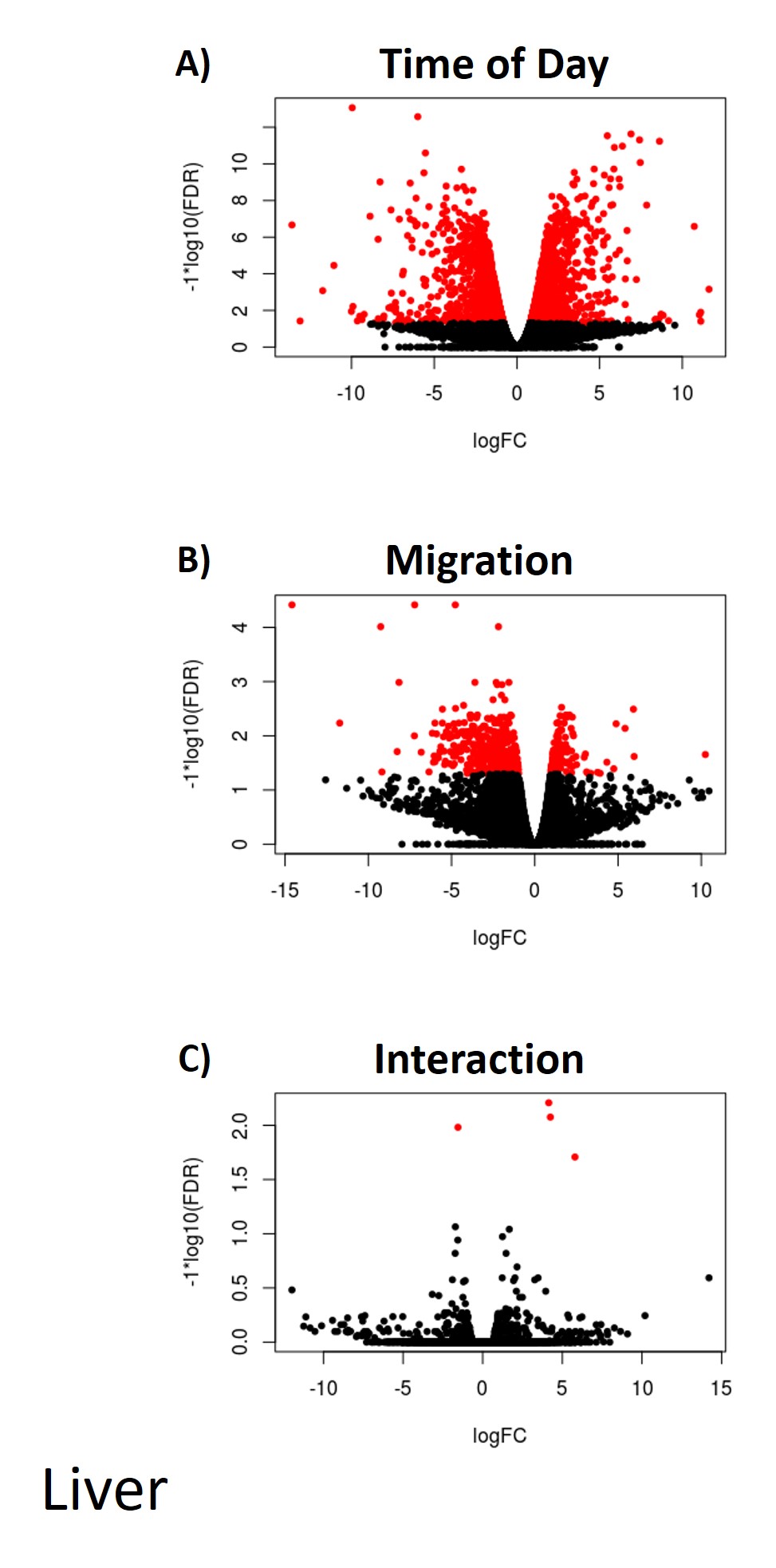


**Figure SI5: Volcano Plots of Differentially Expressed Genes in the Liver.**

Volcano plots of differentially expressed genes in the liver as a factor of the main effect of time of day (A), migratory status (B) or the interaction of time of day x migratory status (C). Black colored points indicate non-significant findings, while red-colored dots are significant at p<0.05.


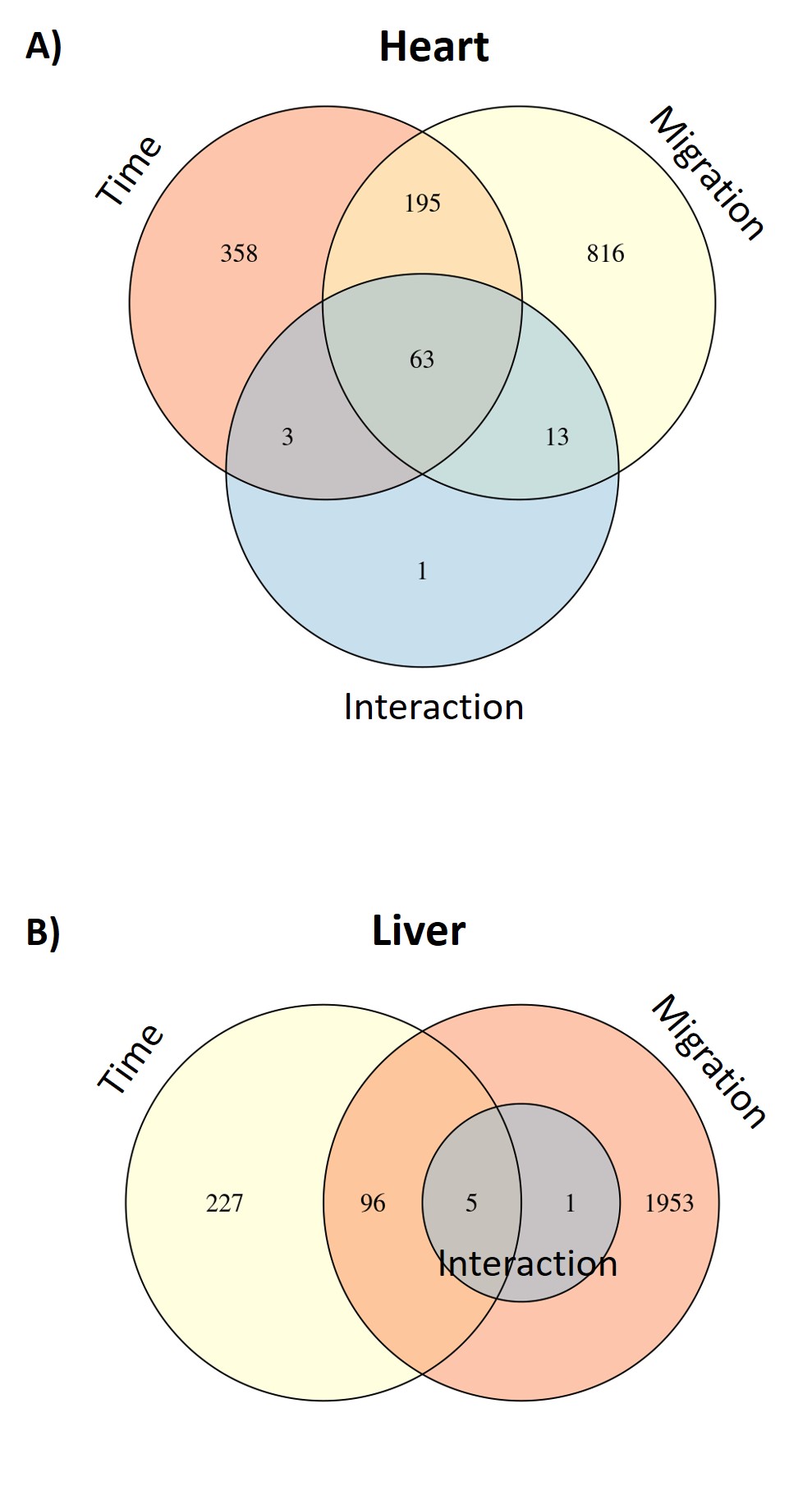


**Figure SI6: Overlap of Differentially Expressed Genes by Tissue and Effect.**

Venn diagrams showing the number of unique and common genes with effects of time of day, migratory status and the time of day x migratory status interaction. Panel A diagrams the data from the heart, and panel B shows the genes from the liver.


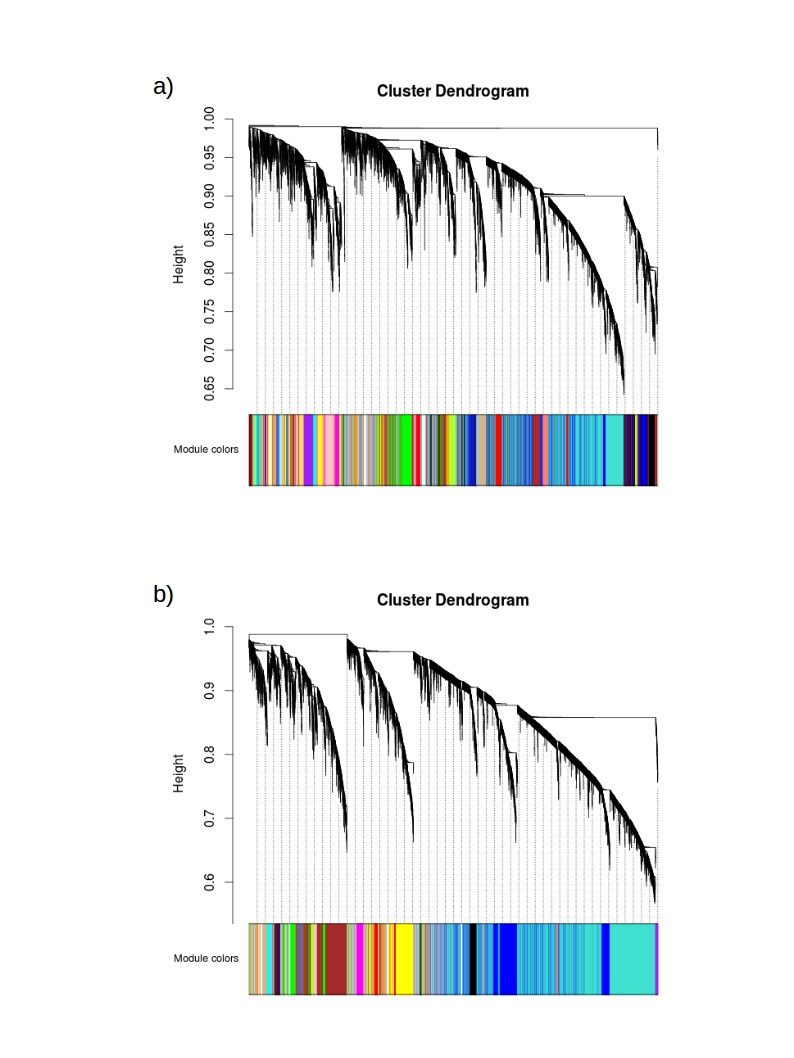


**Figure SI8: WGCNA Network Construction in Heart and Liver**

This dendrogram shows weighted gene co-expression network construction in the heart. Genes are grouped into modules, as denoted by the colored bar along the bottom, based on hierarchical clustering. The y-axis is a measure of dissimilarity based on topological overlap, while each line represents a single gene.
